# Comparison of effects of sitagliptin and voglibose on left ventricular diastolic dysfunction in patients with type 2 diabetes: results of the 3D trial

**DOI:** 10.1186/s12933-015-0242-z

**Published:** 2015-06-19

**Authors:** Hiroki Oe, Kazufumi Nakamura, Hajime Kihara, Kenei Shimada, Shota Fukuda, Tsutomu Takagi, Toru Miyoshi, Kumiko Hirata, Junichi Yoshikawa, Hiroshi Ito, FESC, for Effect of a DPP-4 inhibitor on left ventricular diastolic dysfunction in patients with type 2 diabetes and diabetic cardiomyopathy (3D) study investigators.

**Affiliations:** Center of Ultrasonic Diagnostics, Okayama University Hospital, Okayama, Japan; Department of Cardiovascular Medicine, Okayama University Graduate School of Medicine, Dentistry and Pharmaceutical Sciences, 2-5-1 Shikata-cho, Okayama, 700-8558 Japan; Department of Internal Medicine, Kihara Cardiovascular Clinic, Asahikawa, Japan; Department of Internal Medicine and Cardiology, Osaka City University of Medicine, Osaka, Japan; Department of Medicine, Osaka Ekisaikai Hospital, Osaka, Japan; Takagi Cardiology Clinic, Kyoto, Japan; Department of Cardiovascular Medicine, Wakayama Medical University, Wakayama, Japan; Nishinomiya Watanabe Cardiovascular Center, Nishinomiya, Japan

**Keywords:** Dipeptidyl peptidase 4 (DPP-4) inhibitors, Alpha-glucosidase inhibitor, LV diastolic function

## Abstract

**Background:**

Left ventricular (LV) diastolic dysfunction is frequently observed in patients with type 2 diabetes. Dipeptidyl peptidase-4 inhibitor (DPP-4i) attenuates postprandial hyperglycemia (PPH) and may have cardio-protective effects. It remains unclear whether DPP-4i improves LV diastolic function in patients with type 2 diabetes, and, if so, it is attributable to the attenuation of PPH or to a direct cardiac effect of DPP-4i. We compared the effects of the DPP-4i, sitagliptin, and the alpha-glucosidase inhibitor, voglibose, on LV diastolic function in patients with type 2 diabetes.

**Methods:**

We conducted a prospective, randomized, open-label, multicenter study of 100 diabetic patients with LV diastolic dysfunction. Patients received sitagliptin (50 mg/day) or voglibose (0.6 mg/day). The primary endpoints were changes in the e’ velocity and E/e’ ratio from baseline to 24 weeks later. The secondary efficacy measures included HbA1c, GLP-1, lipid profiles, oxidative stress markers and inflammatory markers.

**Results:**

The study was completed with 40 patients in the sitagliptin group and 40 patients in the voglibose group. There were no significant changes in the e’ velocity and E/e’ ratio from baseline to 24 weeks later in both groups. However, analysis of covariance demonstrated that pioglitazone use is an independent factor associated with changes in the e’ and E/e’ ratio. Among patients not using pioglitazone, e’ increased and the E/e’ ratio decreased in both the sitagliptin and voglibose groups. GLP-1 level increased from baseline to 24 weeks later only in the sitagliptin group (4.8 ± 4.7 vs. 7.3 ± 5.5 pmol/L, *p* < 0.05). The reductions in HbA1c and body weight were significantly greater in the sitagliptin group than in the voglibose group (−0.7 ± 0.6 % vs. −0.3 ± 0.4, *p* < 0.005; −1.3 ± 3.2 kg vs. 0.4 ± 2.8 kg, *p* < 0.05, respectively). There were no changes in lipid profiles and inflammatory markers in both groups.

**Conclusions:**

Our trial showed that sitagliptin reduces HbA1c levels more greatly than voglibose does, but that neither was associated with improvement in the echocardiographic parameters of LV diastolic function in patients with diabetes.

**Trial registration:**

Registered at http://www.umin.ac.jp under UMIN000003784

## Background

Diabetes mellitus is a major risk factor for heart failure (HF), especially HF with preserved ejection fraction (HFPEF) development [1, 2]. Diabetes and HF commonly coexist, and together these conditions are associated with increased morbidity and mortality compared with either condition alone [3–7]. A recent study in Olmsted County, MN, USA, has shown that the prevalence of diabetes in HF patients has increased markedly over time (3.8 % per year) [8]. An angiotensin-converting enzyme inhibitor (ACEi) or an angiotensin receptor blocker (ARB) is effective in treating HF as well as reducing insulin resistance in patients with diabetes [9]. Other therapeutic strategies are lacking the same level of effectiveness. Studies have shown that elevated HbA1c is a marker of increased risk of developing HF and, therefore, poor glycemic control may be causally related to the development of HF [10, 11]. However, the optimal treatment of hyperglycemia in patients with diabetes to reduce the progression of HF has not been well studied.

Recent studies have shown that dipeptidyl peptidase-4 inhibitor (DPP-4i), which increases the circulating glucagon-like peptide-1 (GLP-1) level, attenuates postprandial hyperglycemia and may have cardio-protective effects [12–14]. In a diabetic rat model, Shigeta et al. [15] demonstrated that DPP4 inhibition reverses left ventricular (LV) diastolic dysfunction via membrane-bound DPP4/stromal cell-derived factor-1α-dependent local actions on angiogenesis and circulating DPP4/GLP-1-mediated inotropic actions. Using HF-model rats, dos Santos et al. [16] reported that sitagliptin (40 mg/kg) administered for 6 weeks exhibited a significant improvement in cardiac contraction and reduction in LV end-diastolic pressure and chamber stiffness. Small pilot studies of GLP-1 have shown potential promise in the treatment of HF patients [17, 18]. However, it remains unknown whether DPP-4i improves LV diastolic function in patients with type 2 diabetes, and, if so, whether the effect is attributable to the attenuation of postprandial hyperglycemia or to the direct cardiac effect of DPP-4i.

## Methods

We conducted a randomized, prospective, open-label, multicenter study to compare the effects of sitagliptin, a DPP-4 inhibitor, and voglibose, an alpha GI, on LV diastolic function. The assessment was done by Doppler echocardiography in patients with type 2 diabetes at 13 sites between January 2011 and January 2013.

### Study population

Our study population consisted of outpatients with type 2 diabetes from 20 to 85 years of age with LV diastolic dysfunction (LV ejection fraction >50 %, mitral annular early diastolic velocity (e’) <8 cm/s or the ratio of mitral inflow velocity to e’ velocity (E/e’ ratio) >15). These patients had not achieved the targets for glycemic control with diet, exercise, sulfonylurea, metformin or pioglitazone treatments. We recruited 100 patients with 50 receiving sitagliptin (50 mg/day) treatment and 50 voglibose (0.6 mg/day). The doses of the two drugs used in this study are the recommended therapeutic doses for Japanese patients who are covered by the Japanese National Health Insurance.

The exclusion criteria were: patients being treated with insulin, alpha GI or glinide; and/or exhibited any of the following: type 1 diabetes, HbA1c ≥9.0 % (75 mmol/mol), systolic blood pressure ≥160 mmHg and serum creatinine ≥1.5 mg/dL at baseline, myocardial infarction (MI) or stroke within the previous 24 weeks, significant LV hypertrophy at baseline (LV wall thickness ≥13 mm), atrial fibrillation at baseline, and significant valve diseases (more than or equal to moderately severe valve diseases). The study protocol was approved by the Ethics Committee of Okayama University Graduate School of Medicine, Dentistry, and Pharmaceutical Sciences, and of each hospital. Written informed consent was obtained from all patients before any study procedure.

### Study protocol

**Fig. 1:**
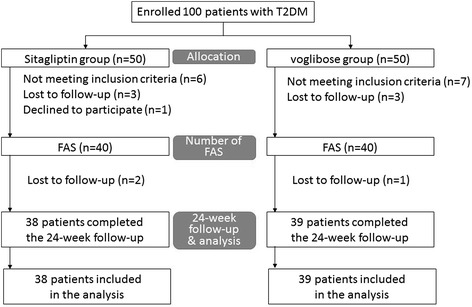
The study’s workflow. Twenty patients were excluded including 6 who were lost to follow-up, 1 who declined to participate and 13 for protocol violation (not meeting inclusion criteria, 12 patients; LVEF < 50 %, 1; under medical treatment with high-dose sulfonylurea). The follow-up study was completed in 77 (77 %) of the patients; 38 received sitagliptin and 39 voglibose

The patients were followed for at least 8 weeks to observe that the treatment goal by diet, exercise, sulfonylurea, metformin or pioglitazone was not being achieved. The patients were prospectively and randomly assigned to additional treatment with either sitagliptin (50 mg/day) or voglibose (0.6 mg/day) for at least 24 weeks (Fig. 1). We chose a stratified block randomization. Stratified block randomization was computer-generated and done by use of a web-based system (Nouvelle Place Inc. [http://www.n-place.co.jp/]). Patients were stratified according to age (≥ or < 65 years old), HbA1c (≥ or < 7 %), e’ (≥ or < 6.0 cm/s), and combined use of thiazolidinediones.

Standard echocardiography was performed at the baseline and after 24 weeks of treatment. From the mitral flow velocity pattern, measurements were taken of peak velocities of E and A waves, the ratio of their peak velocities (E/A ratio) and deceleration time of the E wave. Spectral pulsed-wave Doppler tissue interrogation of longitudinal mitral annular velocity was recorded throughout the cardiac cycle at the septal annulus in the apical four-chamber view. The peaks of myocardial systolic apically directed velocity (s’) and early diastolic velocity (e’) were measured.

Additional exploratory analyses including changes in the chamber dimensions and LV ejection fraction were assessed. LV mass was measured by using the American Society of Echocardiography-recommended formula and the end-systolic left atrial volume was measured by using the ellipsoid model [19]. Both values were indexed with body surface area in m^2^. All echocardiographic studies were done when patients were clinically stable on treatment.

### Measurements of biochemical parameters

The following parameters were measured at baseline and after 24 weeks of treatment: complete blood count, liver function test including measurement of AST, ALT and LDH, renal function test including measurement of BUN, creatinine, Na, K and Cl, HbA1c, gastric inhibitory peptide (GIP), GLP-1, C-peptide, CD34, lipid profile including total cholesterol, triglyceride, and high-density lipoprotein (HDL-C), adiponectin, oxidative stress markers including malondialdehyde-modified low density lipoprotein (MDA-LDL) and urine 8-hydroxy-2′-deoxyguanosine (8-OHdG), inflammatory markers including high-sensitive C-reactive protein (hs-CRP) and pentraxin-3 (PTX-3), and estimated glomerular filtration rate (eGFR). HbA1c levels were measured using high-performance liquid chromatography. The number of CD34+ cells was determined by flow cytometry using fluorescein isothiocyanate-labeled CD45 and phycoerythrin (PE)-labeled CD34 antibodies (BD Biosciences, Franklin Lakes, NJ, USA). eGFR (mL/min/1.73 m^2^) was determined by the modified Modification of Diet and Renal Disease study formula (MDRD) for Japanese: eGFR = 194 × (age-0.287) × (serum creatinine-1.094) × (0.739 if female). Brain natriuretic peptide (BNP) concentrations were measured using a commercially available specific radioimmunoassay for human BNP (Shiono RIA BNP assay kit, Shionogi Co., Ltd., Osaka, Japan), respectively. Antihypertensive, antihyperlipidemic and antidiabetic drugs were not changed and anti-oxidant drugs including vitamin C and E were not added throughout the study period.

### Endpoints

The primary endpoints were changes in e’ and E/e’ ratio from baseline to the end of follow-up. The secondary efficacy measures included changes in glucose, HbA1c, GIP, GLP-1, C-peptide, CD34, lipid profile, oxidative stress markers including MDA-LDL and 8-OHdG, inflammatory markers including hs-CRP and PTX-3 and eGFR, and any adverse events.

### Statistical analysis

All of the results are expressed as mean ± SD or as proportions (%). We assumed that e’ increased by 1.0 cm/s in the sitagliptin group and 0.2 cm/s in the voglibose group with a standard deviation of 1.5 cm/s [20]. A minimum sample size of 57 participants in each group was required to detect statistical differences in e’ with a power of 80 % and α error of 5 %. The effects of sitagliptin and voglibose on LV diastolic function were compared using a paired *t*-test. Differences in age, sex, weight, body mass index and blood pressure were compared using the Student’s *t*-test. Categorical variables were compared using *χ*^2^ test and Fisher’s exact test when appropriate. Differences in secondary efficacy measures between baseline and 24 weeks were compared using a paired *t*-test. A value of p < 0.05 was set as the threshold for significance. The effects of sitagliptin and voglibose on LV diastolic function (e’ and E/e’ ratio) were assessed by analysis of covariance (ANCOVA) after adjustment for covariates that included baseline e’ and E/e’ ratio, age, sitagliptin/voglibose use and concomitant thiazolidinedione use. Multivariate analysis of factors related to changes from baseline to 24 weeks in e’ and E/e’ ratio levels was performed. All analyses were performed using SAS 9.3 (SAS Institute Inc., Cary, NC, USA).

## Results

### Baseline characteristics

One hundred patients (59 men and 41 women, aged 67.1 ± 9.6 years) with type 2 diabetes were enrolled in this study. Fifty patients received sitagliptin (50 mg/day) treatment and 50 patients received voglibose (0.6 mg/day) treatment. Twenty patients were excluded including 7 who were lost to follow-up and 13 for protocol violation (not meeting inclusion criteria, 6 patients;LV ejection fraction <50 %, 1; under medical treatment with high-dose sulfonylurea.). The follow-up study was completed in 80 (80 %) of the patients; 40 received sitagliptin and 40 voglibose (Fig. 1). Baseline clinical characteristics, including age, sex, body mass index and the type of antidiabetic and antihypertensive medication are shown in Table 1. No patients died, developed cardiovascular events or were admitted to the hospital during the study.

**Table 1:** Baseline clinical characteristics

Variable	Sitagliptin n = 40	Voglibose n = 40	*P* value
Age, y	67.8 ± 10.5	66.7 ± 9.8	0.654
Women, n (%)	20(50)	14(35)	0.258
Diabetes duration, months (95 % CI)	48(6-240)	38.5(3-218)	0.145
Body mass index, kg/m2	27.7 ± 4.1	25.7 ± 4.3	0.038
Abdominal girth, cm	88.7 ± 14.3	85.9 ± 15.6	0.469
Current smoking	6(15)	4(10)	0.762
Regular alcohol drinkers	13(33)	17(43)	0.489
NYHA functional class			
Class I, n (%)	17(43)	17(43)	1.000
Class II, n (%)	23(58)	22(55)	
Class III, n (%)	0(0)	1(3)	
Grades of Diastolic Dysfunction			
0	1 (2.6)	3 (7.5)	0.0987
1	31 (79.5)	34 (85.0)	
2	7 (17.9)	3 (7.5)	
Diabetes complication+	7(18)	3(8)	0.311
Diabetic retinopathy	4(10)	2(5)	0.675
Diabetic nephropathy	3(8)	0(0)	0.241
Diabetic neuropathy	2(5)	0(0)	0.494
Hypertension	37(93)	32(80)	0.193
Hyperuricemia	31(78)	31(78)	1.000
Hyperlipidemia	5(13)	4(10)	1.000
Renal disturbance	7(18)	1(3)	0.057
Mean eGFR	74.8 ± 21.7	70.5 ± 15.3	0.304
Cerebrovascular disease	3(8)	1(3)	0.615
Myocardial infarction	5(13)	2(5)	0.432
Peripheral artery disease	3(8)	1(3)	0.615
Anti-diabetic drugs			
Pioglitazone	14(35)	18(45)	0.494
Sulfonylurea	6(15)	3(8)	0.481
Metformin	7(18)	1(3)	0.057
α-GI	1(3)	0(0)	1.000
Antihypertensive drugs			
ARB	28(70)	27(68)	1.000
Calcium channel blocker	26(65)	22(55)	0.494
Diuretics	15(38)	10(25)	0.335
Others	11(28)	10(25)	1.000
ACE-I	2(5)	3(8)	
α-blocker	4(10)	1(3)	
β-blocker	6(15)	4(10)	
αβ-blocker	1(3)	1(3)	
Aldosterone antagonist	2(5)	1(3)	
Antihyperlipidemic drugs			
Statins	24(60)	22(55)	0.821
Fibrate	2(5)	2(5)	1.000
Ezetimibe	5(13)	6(15)	1.000
Eicosapentaenoic acid	5(13)	2(5)	0.432
Antiplatelet agent	10(25)	12(30)	0.803
Nitrates	1(3)	1(3)	1.000
Allopurinol	1(3)	4(10)	0.359
Uricosuric agents	0(0)	1(3)	1.000

Values are means ± SD, number of patients (%), or median (95 % confidencel Interval). The continuous variables were compared using Student’s *t*-test, the categorical variables were compared using Fisher’s exact test, grades of diastolic dysfunction was compared using Cochran-Armitage trend test, and the duration of diabetes was compared using *the Wilcoxon rank sum test*

### The primary endpoint

**Fig. 2:**
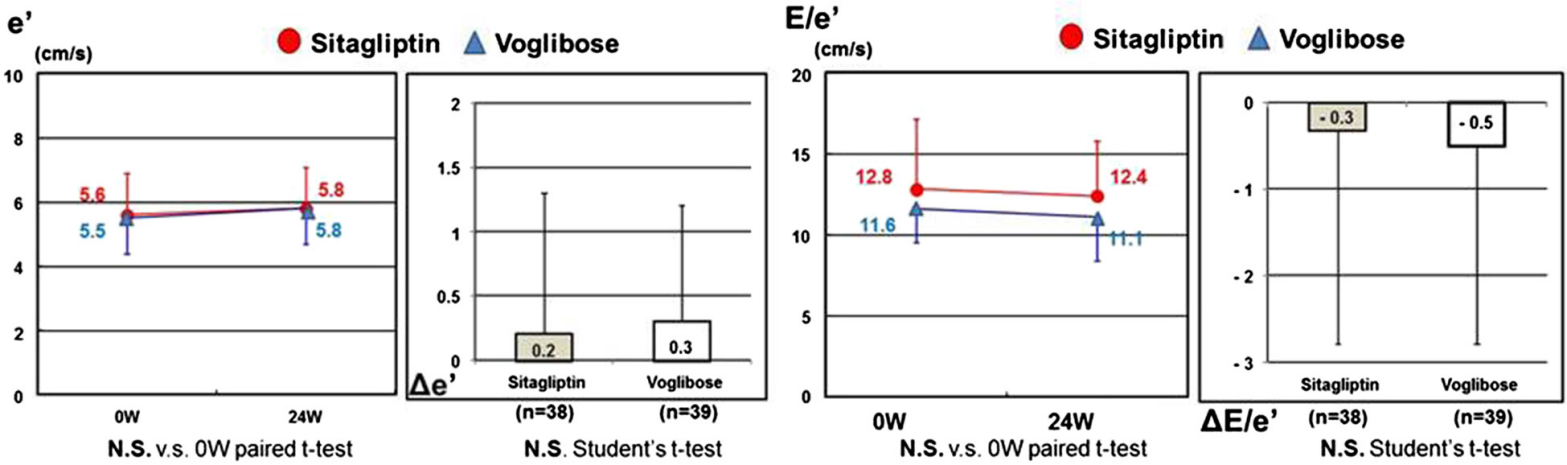
Changes in e’ and E/e’ between baseline and 24 weeks later. The e’ velocity and E/e’ ratio were comparable between sitagliptin and voglibose groups at baseline. The e’ velocity and E/e’ ratio showed no changes between baseline and a mean of 24 weeks later in both groups. There was also no significant difference in the magnitude of the changes in e’ velocity and E/e’ ratio between the two groups. e’, mitral annular early diastolic velocity; E/e’, ratio of mitral inflow velocity to e’ velocity; W, weeks

The e’ velocity and E/e’ ratio were comparable between the sitagliptin and voglibose groups at baseline. The e’ velocity and E/e’ ratio in both groups showed no changes between baseline and after a mean of 24 weeks (e’: sitagliptin +0.2 ± 1.1 cm/s and voglibose +0.3 ± 0.9 cm/s; E/e’: sitagliptin −0.3 ± 2.5 and voglibose −0.5 ± 2.3). There was also no difference in magnitude of the changes in e’ velocity and E/e’ ratio between the two groups (Table 2 and Fig. 2).

**Table 2:** Changes in e’ velocity and E/e’ ratio in the sitagliptin and voglibose group

Variable	Sitagliptin n = 40	Voglibose n = 40	Between-group difference
e’			
Baseline at 0W	5.6 ± 1.3	5.5 ± 1.1	p = 0.664
at 24W	5.8 ± 1.3	5.8 ± 1.1	p = 0.997
Δe’: 24W-0W	0.2 ± 1.1	0.3 ± 0.9	p = 0.702
E/e’			
Baseline at 0W	12.8 ± 4.3	11.6 ± 2.1	p = 0.109
at 24W	12.4 ± 3.4	11.1 ± 2.7	p = 0.060
ΔE/e’: 24W-0W	-0.3 ± 2.5	-0.5 ± 2.3	p = 0.804

Values are means ± SD

*W* weeks

The effects of sitagliptin and voglibose on LV diastolic function (e’ and E/e’ ratio) were assessed by ANCOVA after adjustment for covariates that included baseline e’ and E/e’ ratio, age, sitagliptin/voglibose use and concomitant thiazolidinedione use. ANCOVA demonstrated that pioglitazone use is an independent factor associated with changes in e’ and E/e’ ratio (Table 3). Among patients not using pioglitazone, e' increased and E/e’ ratio decreased in both sitagliptin and voglibose groups (e’: sitagliptin +0.5 ± 1.1 cm/s and voglibose +0.5 ± 0.9 cm/s; E/e’: sitagliptin −1.0 ± 2.7 and voglibose −0.8 ± 2.4).

**Table 3:** Factors affecting e’ and E/e’ at week 24^a^

Dependent variable	Independent variable	Estimate	95 % CI		*P* value
e’ (24W)	e’ (0W)	0.525	0.309	0.742	<0.0001
	Sitagliptin/Voglibose	-0.047	-0.455	0.362	0.821
	Age — yr	-0.028	-0.054	-0.003	0.032
	without/with TZD	0.547	0.130	0.965	0.011
E/e’ (24W)	E/e’ (0W)	0.615	0.471	0.758	<0.0001
	Sitagliptin/Voglibose	0.688	-0.239	1.615	0.144
	Age — yr	0.024	-0.025	0.072	0.334
	without/with TZD	-1.411	-2.339	-0.483	0.003

95 % CIs and *P* values were calculated with the use of analysis of covariance (ANCOVA)

^a^CI denotes confidence interval

Multivariate analysis of factors related to changes from baseline to 24 weeks in e’ and E/e’ ratio levels showed for e’, without/with thiazolidinedione was not statistically significant only if adiponectin was added in the model, and for E/e’, without/with thiazolidinedione was statistically significant for all models, which implies that without/with thiazolidinedione could be an independent factor associated with changes in the E/e’ ratio (Table 4).

**Table 4:** Multivariate analysis of factors related to changes from baseline to week 24 in e’ and E/e’ ratio levels^a^

Model	e’ [cm/s]			E/e’		
	Change from baseline	P value^b^	R2	Change from baseline	P value^b^	R2
	Beta (95 % CI)			Beta (95 % CI)		
Model1—Primary model			0.500			0.609
Baseline	0.525 (0.309 to 0.742)	<.0001		0.615 (0.471 to 0.758)	<.0001	
Sitagliptin/Voglibose	-0.047 (-0.455 to 0.362)	0.8206		0.688 (-0.239 to 1.615)	0.1436	
Age—yr	-0.028 (-0.054 to -0.003)	0.0317		0.024 (-0.025 to 0.072)	0.3335	
Without/with TZD	0.547 (0.130 to 0.965)	0.0109		-1.411 (-2.339 to -0.483)	0.0034	
Model2—Add demographic values^b^			0.522			0.641
Baseline	0.520 (0.298 to 0.742)	<.0001		0.603 (0.456 to 0.750)	<.0001	
Sitagliptin/Voglibose	-0.069 (-0.504 to 0.365)	0.7509		0.683 (-0.271 to 1.636)	0.1576	
Age—yr	-0.025 (-0.052 to 0.002)	0.0739		0.013 (-0.037 to 0.064)	0.5964	
Female/Male	0.243 (-0.195 to 0.681)	0.2718		-1.114 (-2.092 to -0.137)	0.0261	
BMI (0W)	0.033 (-0.019 to 0.084)	0.2140		-0.037 (-0.155 to 0.081)	0.5306	
SBP (0W)	-0.005 (-0.020 to 0.011)	0.5454		-0.002 (-0.036 to 0.031)	0.8917	
Pulse (0W)	-0.003 (-0.025 to 0.019)	0.7882		0.022 (-0.028 to 0.071)	0.3911	
Without/with TZD	0.662 (0.216 to 1.108)	0.0042		-1.732 (-2.711 to -0.753)	0.0007	
Model3—Add complications^b^			0.518			0.626
Baseline	0.487 (0.253 to 0.721)	<.0001		0.584 (0.425 to 0.744)	<.0001	
Sitagliptin/Voglibose	0.018 (-0.426 to 0.462)	0.9362		0.642 (-0.331 to 1.615)	0.1922	
Age—yr	-0.031 (-0.057 to -0.004)	0.0228		0.030 (-0.020 to 0.081)	0.2389	
Diabetes complication	-0.395 (-1.038 to 0.247)	0.2238		0.721 (-0.799 to 2.241)	0.3473	
Hypertension	0.202 (-0.410 to 0.814)	0.5131		-0.527 (-1.908 to 0.854)	0.4491	
Hyperlipidemia	0.219 (-0.317 to 0.755)	0.4177		-0.886 (-2.086 to 0.313)	0.1450	
Renal disturbance	-0.219 (-1.000 to 0.562)	0.5780		0.277 (-1.453 to 2.007)	0.7502	
Without/with TZD	0.511 (0.068 to 0.954)	0.0244		-1.302 (-2.271 to -0.334)	0.0092	
Model4—Add laboratory values^b^			0.525			0.642
Baseline	0.542 (0.314 to 0.770)	<.0001		0.615 (0.469 to 0.761)	<.0001	
Sitagliptin/Voglibose	-0.127 (-0.568 to 0.314)	0.5671		0.669 (-0.314 to 1.651)	0.1790	
Age—yr	-0.023 (-0.053 to 0.006)	0.1220		0.029 (-0.027 to 0.084)	0.3045	
WBC (0W)	-0.000 (-0.000 to 0.000)	0.7030		0.000 (-0.000 to 0.000)	0.2013	
AST (0W)	0.012 (-0.008 to 0.032)	0.2398		0.003 (-0.040 to 0.046)	0.8871	
BUN (0W)	0.009 (-0.045 to 0.063)	0.7380		-0.019 (-0.139 to 0.101)	0.7498	
Glucagon (0W)	-0.001 (-0.012 to 0.010)	0.8296		-0.010 (-0.034 to 0.014)	0.3929	
Adiponectin (0W)	-0.017 (-0.049 to 0.016)	0.3067		-0.030 (-0.102 to 0.041)	0.4020	
IVSTh (0W)	-0.192 (-1.847 to 1.463)	0.8175		-1.323 (-5.000 to 2.353)	0.4749	
Without/with TZD	0.380 (-0.181 to 0.941)	0.1808		-1.675 (-2.916 to -0.434)	0.0089	
Model5—Add adiponectin^c^			0.511			0.620
Baseline	0.516 (0.299 to 0.732)	<.0001		0.623 (0.480 to 0.766)	<.0001	
Sitagliptin/Voglibose	-0.080 (-0.490 to 0.330)	0.6991		0.587 (-0.344 to 1.519)	0.2129	
Age—yr	-0.025 (-0.051 to 0.001)	0.0637		0.033 (-0.016 to 0.083)	0.1858	
Adiponectin (0W)	-0.018 (-0.047 to 0.011)	0.2169		-0.046 (-0.112 to 0.019)	0.1610	
Without/with TZD	0.388 (-0.099 to 0.876)	0.1169		-1.807 (-2.884 to -0.730)	0.0013	
Model6—Remove TZD			0.493			0.560
Baseline	0.480 (0.266 to 0.695)	<.0001		0.624 (0.472 to 0.777)	<.0001	
Sitagliptin/Voglibose	-0.067 (-0.481 to 0.347)	0.7471		0.566 (-0.429 to 1.561)	0.2605	
Age—yr	-0.026 (-0.052 to 0.001)	0.0580		0.025 (-0.028 to 0.078)	0.3505	
Adiponectin (0W)	-0.030 (-0.055 to -0.005)	0.0187		0.010 (-0.050 to 0.070)	0.7331	

^a^The analysis was performed in the full analysis set, with the use of analysis of covariance, WBC denotes white blood cell, AST aspartate transaminase, BUN blood urea nitrogen, IVSTh interventricular septum thickness, and TZD thiazolidinedione

^b^Statistically significant variables (alpha = 0.05) were selected from measured demographics, complications and laboratory variables with the use of two sample t-tests, according to without/with TZD groups in the full analysis set

^c^For e’, without/with TZD was not statistically significant (alpha = 0.05) only if adiponectin was added in the model

### Secondary efficacy measures

**Fig. 3:**
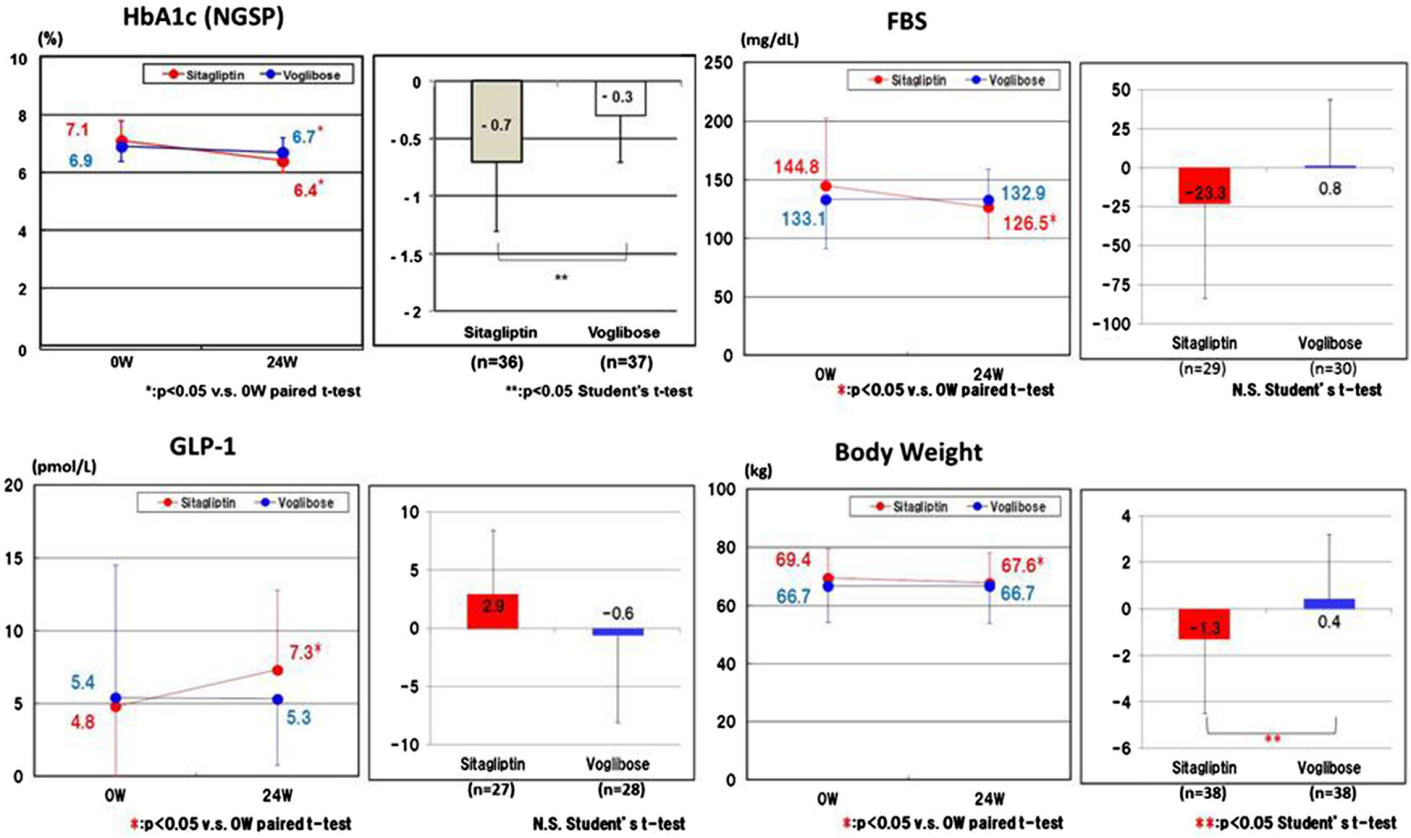
Changes in HbA1c, FBS, GLP-1 and body weight between baseline and 24 weeks later. The decreases in HbA1c and body weight were significantly greater in the sitagliptin group than in the voglibose group. GLP-1 level increased from baseline to 24 weeks later in the sitagliptin group but not in the voglibose group. FBS, fasting blood sugar; GLP-1, glucagon-like peptide-1; HbA1c, hemoglobin A1c; NGSP, National Glycohemoglobin Standardization Program; W, weeks

The GLP-1 level increased from baseline to 24 weeks later in the sitagliptin group but not in the voglibose group (4.8 ± 4.7 pmol/L vs. 7.3 ± 5.5 pmol/L, p < 0.05, respectively). The decreases in HbA1c and body weight were significantly greater in the sitagliptin group than in the voglibose group (−0.7 ± 0.6 % vs. −0.3 ± 0.4 %, p < 0.005 and −1.3 ± 3.2 kg vs. 0.4 ± 2.8 kg, p < 0.05, respectively). There were no significant differences in the changes in C-peptide, lipid profile, oxidative stress marker and inflammatory marker between the two groups (Table 5 and Fig. 3). In only the sitagliptin group, and not the voglibose group, did the systolic blood pressure and LV septal wall thickness decrease 24 weeks later (135 ± 16 mmHg vs. 128 ± 10 mmHg and 0.97 ± 0.15 mm vs. 0.93 ± 0.14 mm, respectively) (Table 6).

**Table 5:** Changes in secondary efficacy measures in the sitagliptin and voglibose group

Variable	Sitagliptin		Voglibose		Between-group difference
	Mean ± SD	*P* value	Mean ± SD	*P* value	*P* value
Glucose					
Baseline at 0W	145 ± 58		133 ± 42		0.304
At 24W	127 ± 26		133 ± 27		0.354
24W-0W	-23 ± 60	0.047	1 ± 43	0.916	0.083
HbA1c					
Baseline at 0W	7.1 ± 0.7		6.9 ± 0.5		0.273
At 24W	6.4 ± 0.4		6.7 ± 0.5		0.026
24W-0W	-0.7 ± 0.6	<0.0001	-0.3 ± 0.4	0.0004	0.002
GIP					
Baseline at 0W	191 ± 214		159 ± 211		0.517
At 24W	149 ± 161		162 ± 148		0.759
24W-0W	-21 ± 226	0.314	0.6 ± 1.6	0.041	0.749
CD34					
Baseline at 0W	1.1 ± 0.7		0.9 ± 0.6		0.239
At 24W	1.1 ± 0.5		0.9 ± 0.7		0.160
24W-0W	0.1 ± 0.5	0.435	0.0 ± 0.5	0.772	0.430
Total cholesterol					
Baseline at 0W	184 ± 27		185 ± 33		0.944
At 24W	178 ± 29		183 ± 37		0.522
24W-0W	-3 ± 30	0.603	0 ± 29	0.991	0.709
Triglyceride					
Baseline at 0W	155 ± 107		141 ± 74		0.486
At 24W	132 ± 62		127 ± 72		0.803
24W-0W	-15 ± 94	0.391	-13 ± 52	0.190	0.905
HDL-C					
Baseline at 0W	51 ± 12		56 ± 16		0.106
At 24W	52 ± 14		53 ± 15		0.930
24W-0W	0.8 ± 7.9	0.551	-3.6 ± 8.2	0.010	0.021
Adiponectin					
Baseline at 0W	11.0 ± 8.3		13.3 ± 8.7		0.222
At 24W	11.7 ± 8.8		12.9 ± 7.3		0.528
24W-0W	0.5 ± 1.8	0.092	-0.3 ± 2.9	0.539	0.150
MDA-LDL					
Baseline at 0W	106 ± 34		110 ± 37		0.658
At 24W	113 ± 34		109 ± 36		0.591
24W-0W	9 ± 32	0.118	1 ± 38	0.860	0.364
8-OHdG					
Baseline at 0W	11.7 ± 7.9		13.5 ± 10.0		0.392
At 24W	11.4 ± 7.4		14.8 ± 14.6		0.221
24W-0W	-0.2 ± 10.0	0.903	1.5 ± 16.9	0.596	0.605
hs-CRP					
Baseline at 0W	3869 ± 9072		1358 ± 2511		0.099
At 24W	1933 ± 5101		661 ± 691		0.147
24W-0W	-1929 ± 6798	0.098	-380 ± 1186	0.059	0.186
PTX-3					
Baseline at 0W	1.8 ± 1.0		2.4 ± 2.7		0.206
At 24W	1.5 ± 0.8		1.6 ± 0.8		0.557
24W-0W	-0.3 ± 0.7	0.006	-0.3 ± 1.2	0.104	0.974
e-GFR					
Baseline at 0W	75 ± 22		71 ± 15		0.304
At 24W	69 ± 19		72 ± 16		0.411
24W-0W	-5 ± 12	0.014	0 ± 9	0.903	0.036
BNP, pg/ml					
Baseline at 0W	39 ± 48		34 ± 35		0.600
At 24W	40 ± 41		28 ± 24		0.160
24W-0W	1 ± 40	0.923	-5 ± 22	0.164	0.443

*GIP* gastric inhibitory peptide, *GLP-1* glucagon-like peptide-1, *CD34* C-peptide, *MDA-LDL* malondialdehyde-modified low density lipoprotein, *8-OHdG* 8-hydroxy-2′-deoxyguanosine, *hs-CRP* high-sensitive C-reactive protein, *PTX-3*pentraxin-3, *eGFR* estimated glomerular filtration rate, *BNP* B-type natriuretic peptide

**Table 6:** Changes in hemodynamic and echocardiographic parameters in the sitagliptin and voglibose group

Variable	Sitagliptin		Voglibose		Between-group difference
	Mean ± SD	*P* value	Mean ± SD	*P* value	*P* value
SBP, mmHg					
Baseline at 0W	135 ± 16		131 ± 13		0.180
At 24W	128 ± 10		129 ± 15		0.855
24W-0W	-7 ± 17	0.022	-2 ± 15	0.496	0.184
DBP, mmHg					
Baseline at 0W	74 ± 13		75 ± 10		0.818
At 24W	71 ± 10		75 ± 11		0.108
24W-0W	-2 ± 14	0.344	1 ± 10	0.779	0.349
Pulse, b.p.m.					
Baseline at 0W	71 ± 11		71 ± 10		0.903
At 24W	69 ± 12		71 ± 11		0.483
24W-0W	-1 ± 12	0.653	0 ± 12	0.816	0.625
Body weight					
Baseline at 0W	69 ± 10		67 ± 12		0.294
At 24W	68 ± 11		67 ± 13		0.736
24W-0W	-1.3 ± 3.2	0.018	0.4 ± 2.8	0.433	0.020
a’, cm/s					
Baseline at 0W	8.4 ± 1.6		9.0 ± 1.6		0.110
At 24W	8.1 ± 1.8		9.0 ± 1.6		0.031
24W-0W	-0.3 ± 1.7	0.316	0.2 ± 1.4	0.468	0.214
s’, cm/s					
Baseline at 0W	6.6 ± 1.1		7.2 ± 1.2		0.019
At 24W	6.8 ± 1.4		7.0 ± 1.1		0.609
24W-0W	0.2 ± 1.4	0.398	-0.2 ± 1.4	0.412	0.237
Dct					
Baseline at 0W	234 ± 41		237 ± 39		0.769
At 24W	236 ± 42		233 ± 43		0.765
24W-0W	1 ± 41	0.885	-4 ± 35	0.525	0.604
E velocity, m/s					
Baseline at 0W	68 ± 16		62 ± 11		0.048
At 24W	69 ± 14		63 ± 14		0.047
24W-0W	1 ± 10	0.569	0 ± 12	0.879	0.803
A velocity, m/s					
Baseline at 0W	82 ± 19		79 ± 18		0.421
At 24W	80 ± 19		74 ± 14		0.150
24W-0W	-1 ± 13	0.537	-4 ± 10	0.020	0.299
E/A ratio					
Baseline at 0W	0.9 ± 0.3		0.8 ± 0.2		0.424
At 24W	0.9 ± 0.2		0.9 ± 0.3		0.668
24W-0W	0.0 ± 0.2	0.402	0.0 ± 0.2	0.157	0.651
Septal thickness, mm					
Baseline at 0W	1.0 ± 0.2		0.9 ± 0.1		0.069
At 24W	0.9 ± 0.1		0.9 ± 0.1		0.502
24W-0W	0.0 ± 0.1	0.015	0.0 ± 0.1	0.876	0.083
LVEF, %					
Baseline at 0W	68 ± 7		71 ± 7		0.209
At 24W	68 ± 6		66 ± 9		0.179
24W-0W	0 ± 8	0.967	-5 ± 8	0.001	0.013
LVMI, g/m2					
Baseline at 0W	93 ± 20		85 ± 18		0.085
At 24W	90 ± 17		86 ± 18		0.239
24W-0W	-3 ± 13	0.195	1 ± 14	0.551	0.183
LA volume index, ml/m2					
Baseline at 0W	27.7 ± 5.9		26.1 ± 7.6		0.346
At 24W	27.7 ± 7.5		26.6 ± 6.3		0.508
24W-0W	0.3 ± 5.0	0.734	0.8 ± 5.6	0.448	0.733
Urine albumin, mg/g/Cr					
Baseline at 0W	37 ± 66		31 ± 41		0.603
At 24W	39 ± 94		28 ± 50		0.533
24W-0W	-1 ± 50	0.889	-5 ± 30	0.339	0.723
BUN					
Baseline at 0W	17 ± 5		16 ± 5		0.760
At 24W	16 ± 3		16 ± 4		0.824
24W-0W	0 ± 3	0.759	0 ± 4	0.740	0.959

### Adverse events

Adverse events were observed in 2 patients in the sitagliptin group (hypoglycemia and liver dysfunction) and 4 patients in the voglibose group (diarrhea, edema and gastrointestinal symptoms) (Table 7). Mild hypoglycemia of a patient in the sitagliptin group improved rapidly with dose reduction of sulfonylurea. Hospitalizations because of HF or cardiovascular events were not observed during the study period.

**Table 7:** Adverse events

Events	Sitagliptin	Voglibose
	n = 40	n = 40
	2 cases (5.0)	4 cases (10.0)
Hypoglycemia	1 (2.5)	0 (0.0)
Diarrhea	0 (0.0)	4 (10.0)
Edema	0 (0.0)	3 (7.5)
Gastrointestinal symptom	0 (0.0)	2 (5.0)
Liver dysfunction	1 (2.5)	0 (0.0)
Total events	2	9

Number of cases (%) is shown

## Discussion

This study is the first randomized trial to compare the impact of glycemic control with sitagliptin and voglibose on LV diastolic function. Our study demonstrated that both sitagliptin and voglibose reduce HbA1c, but the magnitude of reduction of HbA1c was greater in the sitagliptin group and had a greater increase in the GLP-1 level. However, despite the improved glycemic control, the e’ velocity and E/e’ ratio did not show significant improvement after 24-week treatment with sitagliptin or voglibose. There was also no significant difference in the change in the e’ velocity and E/e’ ratio between the two groups.

The plasma BNP level, New York Heart Association (NYHA) functional class and the grades of diastolic function (ASE/EAE) did not show significant improvement in either group. Therefore, we conclude that sitagliptin and voglibose had no impact on e’ velocity or E/e’ ratio in patients with type 2 diabetes and impaired diastolic function.

Elevated HbA1c has been a marker of increased risk of developing HF in patients with diabetes [10, 11]. Poor glycemic control and associated hyperglycemia may be causally related to the development of HF by two mechanisms: (i) through promotion of atherosclerosis and the ensuing coronary artery disease [21] and (ii) by development of a specific diabetic cardiomyopathy by direct damage to the heart muscle [4, 5]. However, studies examining treatment strategies of intensive glucose control, such as the UKPDS and the more recently completed ACCORD and ADVANCE studies have not shown statistically significant reductions in HF events in patients assigned to more intensive glucose control strategies when compared with those assigned to standard therapy [22–24]. In our study, the blood glucose level was lowered with sitagliptin and voglibose though the reduction of HbA1c was greater in the sitagliptin group, which also had an associated greater increase in the GLP-1 level. Reduction of body weight and systolic blood pressure was also found in the sitagliptin group. These apparently beneficial changes were not, however, associated with an increase in e’ velocity or with a reduction in E/e’ ratio in either groups.

Recently, the SAVOR-TIMI 53 trial showed that DPP-4i may increase the risk of HF [25], which is of concern. In this trial, more patients in the saxagliptin group than in the placebo group were hospitalized for HF (3.5 % vs. 2.8 %, according to the 2-year Kaplan–Meier estimates; hazard ratio 1.27, 95 % CI = 1.07–1.51; p = 0.007). However, our results indicate that the BNP value, e’ velocity and E/e’ ratio do not change during sitagliptin use and, thus, sitagliptin may not be associated with an increased risk of HF or other serious adverse events. Therefore, we can conclude that both sitagliptin and voglibose can be safely used for patients with type 2 diabetes without an increased risk of worsening LV diastolic function.

Although thiazolidinediones activate the peroxisome proliferator-activated receptor γ (PPARγ) system and improve insulin sensitivity to reduce cardiovascular events [26], they are reported to be associated with an increased risk of HF by augmentation of fluid retention [27]. Interestingly, our data demonstrated that pioglitazone is an independent factor that affects the temporal changes in the e’ velocity and E/e’ ratio. The e’ velocity significantly increased in both sitagliptin and voglibose groups if the patients who received pioglitazone were excluded. By contrast, the patients who used pioglitazone showed decreased e’ velocity and increased E/e’ ratio at the follow-up assessment. These data indicate that sitagliptin and voglibose might improve LV diastolic function by themselves, and that pioglitazone may attenuate the beneficial impact of these drugs on LV diastolic function. However, it is uncertain whether pioglitazone may worsen HF, because no patient who used pioglitazone was hospitalized with HF.

An excessive activity of circulating DPP4 was also found to be independently associated with subclinical LV dysfunction in T2DM patients [28]. Decreased adiponectin levels were associated with LV diastolic dysfunction in patients with known or suspected coronary artery disease [29]. Hibuse et al. reported that serum adiponectin level was elevated after a three-month treatment with sitagliptin [30], and that abenefical effect of sitagliptin on LV diastolic dysfunction is expected. However, there was no significant increase in serum adiponectin level (0.5 ± 1.8, p = 0.092) after 24 weeks of treatment with sitagliptin in this study.

Sitagliptin was associated with an increase in GLP-1, but voglibose was not. Increased GLP-1 may augment diuresis and natriuresis by inhibiting sodium reabsorption from the proximal renal tubule [31]. GLP-1 induces an endothelium-dependent vasorelaxation that is dependent on nitric oxide generation, and this may contribute to reduced peripheral vascular resistance [32]. Clinical and experimental studies have shown that DPP-4i has a moderate blood pressure lowering effect [33]. In this study, sitagliptin significantly lowered SBP, but voglibose did not (135 ± 16 mmHg vs. 128 ± 10 mmHg, respectively). There are many studies that demonstrate that reduced systolic blood pressure is associated with regression of LV mass, an increase in e’ velocity and a reduction of HF events [34]. Sitagliptin significantly decreased septal wall thickness in our study, but did not decrease LVMI after the 24-week treatment. In the LV hypertrophy rat model induced by isoproterenol infusion, we previously demonstrated that vildagliptin treatment was associated with a reduction in LV mass compared with the control group [35]. However, the duration of exposure to the study drugs may not have been long enough to reverse the effects of years of diastolic dysfunction processes in patients. The present study does not exclude the possibility of either benefit or increased risk with a longer duration of sitagliptin therapy.

### Owing to the limited sample size, this study is statistically underpowered

However, there was almost no difference (much less than 0.8 cm/s; assumption) of the change in e’ in the estimated value between the two groups in this study. So, even if the target number of cases had been achieved, based on current datasets, it would be unlikely that a statistically significant difference would have been obtained.

There are few data about the effect of anti-diabetic drugs on LV diastolic dysfunction. von Bibra et al. reported that 16 weeks of treatment with rosiglitazone improved LV diastolic dysfunction evaluated by Doppler echocardiography (e’; 7.9 cm/s → 8.9 cm/s, Δe’ = 1.0 cm/s) [20]. However, it remains unknown whether and how anti-diabetic drugs including DPP-4i improve LV diastolic function in patients with type 2 diabetes for now. Further investigation is needed in this area.

We evaluated diastolic function by measuring the e’ velocity and the E/e’ ratio at the septal side of the mitral annulus. Nagueh et al. reported that it is preferable to use the average e’ velocity obtained from the septal and lateral sides of the mitral annulus. We evaluated the changes of e’ and the E/e’ (24 W-0 W) in this study, and our study population excluded patients with LV ejection fraction < 50 % and myocardial infarction within the previous 24 weeks, thus single-site (septal e’) measurements could be applicable.

### Limitations

We acknowledge several limitations in the present study. First, although this study was multicenter and randomized, the drug participation was open-label. Therefore, we used a biomarker, e’ velocity, as the primary endpoint. Secondly, we did not measure plasma DPP-4 activity. The study of dos Santos et al. [16] reported that positive correlations were observed between plasma DPP-4 activity and LV end-diastolic pressure and lung congestion in rats.

We did not know whether 50 mg sitagliptin could successfully inhibit plasma DPP-4 activity. We used 50 mg of sitagliptin, which is a standard starting dosage for this medication in Japan, however, outside of Japan, double this dose, i.e. 100 mg of sitagliptin, is generally used. A meta-analysis revealed that, despite the smaller sitagliptin dose of only 50 mg, a greater HbA1c reduction (−0.99 % versus placebo) was observed in Japanese than in non-Japanese patients [36].

Thirdly, because of the small number of patients, we could not assess whether sitagliptin or pioglitazone can attenuate HF events in patients, as this study is statistically underpowered.

Fourth, 6-month treatment might be too short to improve the e’ velocity or E/e’ ratio. Fifth, echocardiographic studies were performed at each institution by experienced physicians or sonographers and the results were sent to the study center for analysis. These processes are close to those pertaining to real, clinical situations, and diastolic function echocardiographic parameters were supposed to be highly reproducible if performed by experts [37], but inter-institution variability in the examinations may exist and may have influenced the results of this study.

To estimate the inter-institution variability is difficult because the number of cases per institution were too low to evaluate it.

Finally, selection criteria of this study population are strict. It is because we wanted to assess the net effect of DPP inhibitor on LV diastolic function in this study. It is necessary to note whether your patient corresponds to such a patient population or not.

## Conclusion

Our trial showed that sitagliptin reduces HbA1c levels more greatly than voglibose does, but that neither was associated with improvement in the echocardiographic parameters of LV diastolic function in patients with diabetes.

## Abbreviations

BMI: Body mass index
BNP: Brain natriuretic peptide
DPP-4: Dipeptidyl peptidase IV
eGFR: Estimated glomerular filtration rate
FBS: Fasting blood sugar
GIP: Gastric inhibitory peptide
GLP-1: Glucagon-like peptide-1
GI: Glucosidase inhibitor
HF: Heart failure
hs-CRP: High-sensitivity C-reactive protein
8-OHdG: 8-hydroxy-2′-deoxyguanosine
IGT: Impaired glucose tolerance
MDA-LDL: Malondialdehyde-modified low density lipoprotein
NGSP: National glycohemoglobin standardization Program
NYHA: New York heart association
PTX-3: Pentraxin-3
TTE: Trans-thoracic echocardiography
TZD: Thiazolidinedione
